# Supplementary material for: BRCA1/2 Variants and Metabolic Factors: Results From a Cohort of Italian Female Carriers
Source: Cancers (Basel). 2020 Nov 30;12(12):3584. doi: 10.3390/cancers12123584 (PMC7761428; doi:10.3390/cancers12123584)
Supplement: Supplementary file 1 [file cancers-12-03584-s001.pdf]

BRCA1/2 Variants and Metabolic Factors: Results From a Cohort of Italian Female Carriers

Andreina Oliverio, Eleonora Bruno, Mara Colombo, Angelo Paradiso, Stefania Tommasi, Antonella Daniele, Daniela Andreina Terribile, Stefano Magno, Donatella Guarino, Siranoush Manoukian, Bernard Peissel and Paolo Radice and Patrizia Pasanisi

Supplementary Table S1. The table reports the distribution of metabolic characteristics in 438 *BRCA1* and *BRCA2* women.

Table S1. Metabolic characteristics by *BRCA1/2* in 438 female carriers.

	BRCA1 (269)	*BRCA2* (169)
**Weight (Kg)**	64.9 ± 13.8	64.2 ± 13.8
**BMI (kg/m²)**	24.8 ± 5.4	24.4 ± 5.1
**Waist circumferences (cm)**	78.7 ± 12.2	78.5 ± 14.5
**Hip circumferences (cm)**	100.4 ± 10.2	99.6 ± 10.0
**Waist to Height Ratio (cm/cm)**	0.49 ± 0.1	0.49 ± 0.1
**Fat Mass (%)**	31.5 ± 8.0	31.1 ± 8.5
**Systolic pressure (mmHg)**	125.3 ± 16.9	124.5 ± 15.6
**Diastolic pressure (mmHg)**	81.9 ± 10.8	80.9 ± 10.7
**Glycemia (mg/dL)**	101.7 ± 21.7	99.7 ± 23.8
**Total cholesterol (mg/dL)**	201.5 ± 39.6	196.8 ± 39.1
**HDL cholesterol (mg/dL)**	68.2 ± 16.2	67.2 ± 18.1
**LDL cholesterol (mg/dL)**	120.1 ± 35.8	116.2 ± 37.5
**Triglycerides (mg/dL)**	106.1 ± 72.7	100.2 ± 50.0
**IGF-I (ng/mL)**	180.0 ± 66.5	176.4± 68.1
**Insulin (μIU/ml)**	20.7 ± 18.5	20.1 ± 18.6

*p values were established using Student's t test.
